# CytoLNCpred-a computational method for predicting cytoplasm associated long non-coding RNAs in 15 cell-lines

**DOI:** 10.3389/fbinf.2025.1585794

**Published:** 2025-05-26

**Authors:** Shubham Choudhury, Naman Kumar Mehta, Gajendra P. S. Raghava

**Affiliations:** Department of Computational Biology, Indraprastha Institute of Information Technology, New Delhi, India

**Keywords:** lncRNA, cytoplasm localization, machine learning, DNABert-2, cell-line specific localization

## Abstract

The function of long non-coding RNA (lncRNA) is largely determined by its specific location within a cell. Previous methods have used noisy datasets, including mRNA transcripts in tools intended for lncRNAs, and excluded lncRNAs lacking significant differential localization between the cytoplasm and nucleus. In order to overcome these shortcomings, a method has been developed for predicting cytoplasm-associated lncRNAs in 15 human cell-lines, identifying which lncRNAs are more abundant in the cytoplasm compared to the nucleus. All models in this study were trained using five-fold cross validation and tested on an validation dataset. Initially, we developed machine and deep learning based models using traditional features like composition and correlation. Using composition and correlation based features, machine learning algorithms achieved an average AUC of 0.7049 and 0.7089, respectively for 15 cell-lines. Secondly, we developed machine based models developed using embedding features obtained from the large language model DNABERT-2. The average AUC for all the cell-lines achieved by this approach was 0.665. Subsequently, we also fine-tuned DNABERT-2 on our training dataset and evaluated the fine-tuned DNABERT-2 model on the validation dataset. The fine-tuned DNABERT-2 model achieved an average AUC of 0.6336. Correlation-based features combined with ML algorithms outperform LLM-based models, in the case of predicting differential lncRNA localization. These cell-line specific models as well as web-based service are available to the public from our web server (https://webs.iiitd.edu.in/raghava/cytolncpred/).

## Highlights

• Prediction of cytoplasm-associated lncRNAs in 15 human cell lines

• Machine learning using composition and correlation features

• DNABERT-2 embeddings for lncRNA localization prediction

• Correlation-based models outperform LLM-based models

• Web server and models available for public use

## Introduction

The rapidly expanding field of non-coding RNAs has revolutionized our understanding of gene regulation and cell biology. Among the diverse classes of non-coding RNAs, long non-coding RNAs (lncRNAs) have attracted significant attention due to their ability to regulate gene expression at various levels. Initially dismissed as transcriptional noise, lncRNAs have emerged as critical players in cellular processes, including development, differentiation, and disease progression (Statello et al., 2021). To fully comprehend the functional roles of lncRNAs, it is imperative to investigate their subcellular localization. lncRNAs have distinct functions in the nucleus and cytoplasm, influencing transcriptional and posttranscriptional processes. In the nucleus, lncRNAs regulate gene expression and chromatin organization, while in the cytoplasm, they participate in signal transduction and translation. Some lncRNAs exhibit dual localization and functional diversification, reflecting their adaptability to different subcellular environments (Miao et al., 2019; Aillaud and Schulte, 2020; Mattick et al., 2023).

In recent years, extensive research efforts have been focused on deciphering the subcellular localization of lncRNAs. Various experimental approaches, such as fluorescence *in situ* hybridization (FISH) (Chang et al., 2023), RNA sequencing (RNA-seq) (Mayer and Churchman, 2017), and fractionation techniques (Miao et al., 2019), have been employed to identify the subcellular localization patterns of lncRNAs. These studies have revealed that lncRNAs can be localized in different cellular compartments, including the nucleus, cytoplasm, nucleolus, and specific subcellular structures. The subcellular localization of lncRNAs is often associated with their biological functions. For instance, nuclear-localized lncRNAs are frequently involved in transcriptional regulation, chromatin remodeling, and epigenetic modifications. Cytoplasmic lncRNAs, on the other hand, can interact with proteins or act as competitive endogenous RNAs (ceRNAs) to regulate gene expression post-transcriptionally (Bridges et al., 2021). However, most of these methods are expensive to perform and require highly specialized instrumentation.

Advancements in computational methods and machine learning approaches have further facilitated the prediction of lncRNA subcellular localization. These methods leverage various features, such as sequence composition, secondary structure, and evolutionary conservation, to predict the subcellular localization of lncRNAs with high accuracy. Several computational methods have been proposed for predicting lncRNA subcellular localization. Sequence-based methods rely on the nucleotide composition of the lncRNA. They utilize features such as k-mer frequency, nucleotide composition, and sequence motifs. However, these methods are trained on datasets that are not unique to humans, and they do not account for the variation in the subcellular localization of lncRNA in different cells.

**FIGURE 1:**
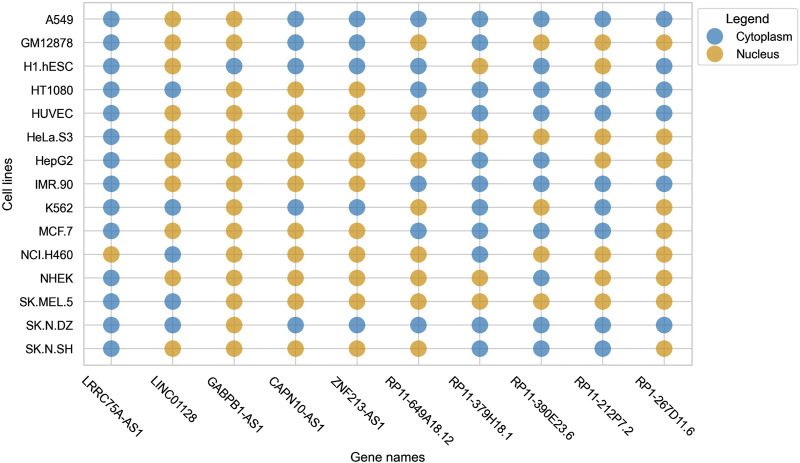
Bubble plot indicating the variability of localization of a single lncRNA across multiple cell-lines.

Cell-line specific subcellular localization gains prominence due to the variability (in terms of subcellular localization) that lncRNAs exhibit within different cell-lines. This was reported by Lin et al. in lncLocator 2.0, where it was observed that a single lncRNA had different localization in different cell-lines (Lin et al., 2021). We observed a similar trend in our dataset, where some lncRNAs were found to be localized in the nucleus for some cell-lines but were localizing to the cytoplasm in some other cell-lines. This pattern can be seen clearly in Figure 1.

lncLocator 2.0 is a cell-line-specific subcellular localization predictor that employs an interpretable deep-learning approach (Lin et al., 2021). TACOS, also a cell-line-specific subcellular localization predictor, uses tree-based algorithms along with various sequence compositional and physicochemical features (Jeon et al., 2022). Among all the existing computational methods, only lncLocator 2.0 and TACOS are designed to predict subcellular localization specific to different cell-lines. The primary issue with these methods is that the datasets used to develop these methods have not been properly filtered. Specifically, these methods have included mRNA sequences in their datasets, which can lead to inaccurate predictions. Additionally, the datasets have eliminated lncRNAs with an absolute fold-change less than 2, which can result in the failure to predict the subcellular location of lncRNAs with borderline concentration differences between locations.

To address the limitations of existing methods in a comprehensive manner, we have developed CytoLNCpred. In this study, we aimed to enhance the prediction accuracy compared to current tools, which have significant room for improvement. Furthermore, we have cleaned the dataset and adhered to industry standards to validate the performance of our method. In CytoLNCpred, a machine learning model trained using correlation-based features demonstrated significantly better performance on the validation dataset compared to existing tools.

## Materials and methods

**FIGURE 2:**
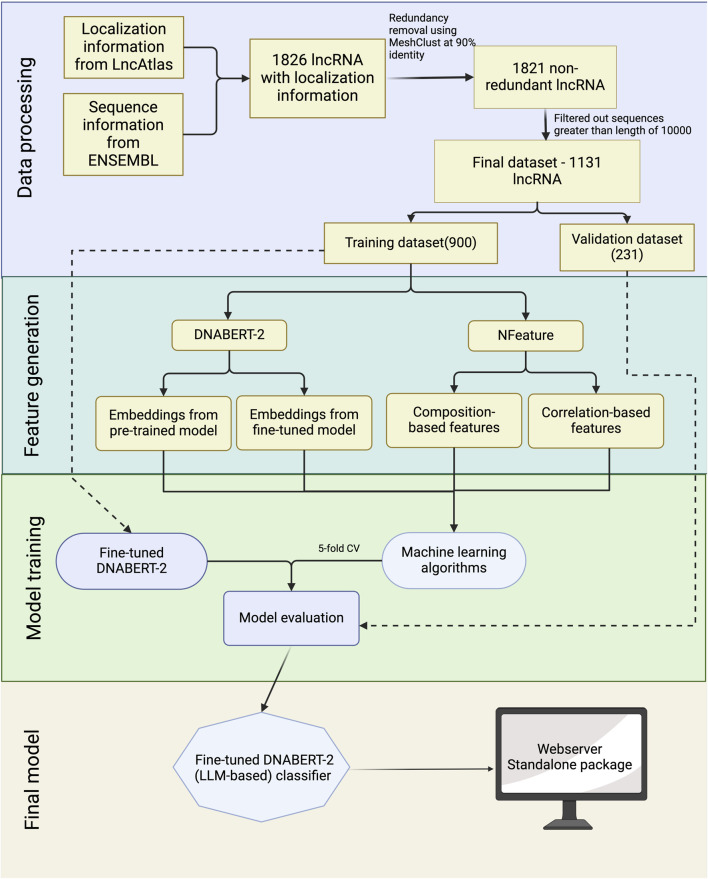
Overall architecture of CytoLNCpred.

To aid in the development of a prediction model for lncRNA subcellular localization, we’ve designed a workflow diagram, depicted in Figure 2. The comprehensive details of each phase in this workflow are outlined in the subsequent sections.

### Dataset creation

In this study, we have selected lncAtlas for acquiring cell-line specific subcellular localization information. lncAtlas is a comprehensive resource of lncRNA localization in human cells based on RNA-sequencing data sets (Mas-Ponte et al., 2017). lncAtlas contains a wide array of information, including Cytoplasm to Nucleus Relative Concentration Index (CNRCI), which we have utilized in our method. CNRCI is defined as the log2-transformed ratio of RPKM (Reads Per Kilobase per Million mapped reads) in two samples, in this case - the cytoplasm and nucleus. It is calculated as follows

$$CNRCI=\log_{2}\left(\frac{Cytoplasmic\;expression\;\left(FPKM\right)}{Nuclear\;expression\;\left(FPKM\right)}\right)$$

**FIGURE 3:**
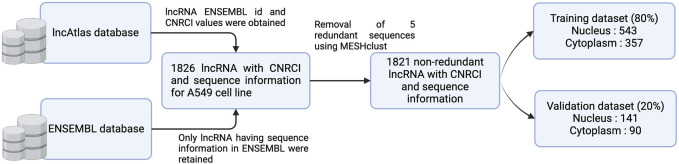
Graphical overview of the process of dataset creation.

Sequence information for the lncRNAs was obtained from ENSEMBL database (version 112) and lncRNAs with no sequence were dropped. In order to modify the dataset for a classification problem, we assigned sequences having CNRCI value greater than 0 as Cytoplasm and those having CNRCI value less than 0 were assigned as Nucleus. Redundancy was removed using MeshClust (Girgis, 2022), using a sequence similarity of 90%. Figure 3 graphically depicts how the training and validation datasets were created.

Further, we used sequences up to the length of 10,000 nucleotides only, as the longer lncRNA were misleading for the machine learning models and computationally very expensive when large language models were involved. The summary of the dataset used for each cell line is provided in Table 1.

**TABLE 1:** Detailed summary of the dataset used in the study, including the total number of samples for each cell-line in the source database and the final non-redundant dataset.

Cell-lines	Original	Non-redundant dataset	Filtering out sequences >10,000 length	Training dataset (complete)	Training dataset (nucleus)	Training dataset (cytoplasm)	Validation dataset (complete)	Validation dataset (nucleus)	Validation dataset (cytoplasm)
	1826	1821	1131	904	547	357	227	137	90
H1.hESC	4194	4178	2552	2041	1224	817	511	307	204
HeLa.S3	1142	1141	703	562	470	92	141	118	23
HepG2	1703	1699	1029	823	598	225	206	150	56
HT1080	1183	1180	690	552	314	238	138	78	60
HUVEC	1870	1859	1137	909	659	250	228	165	63
MCF.7	2714	2703	1702	1361	1028	333	341	257	84
NCI.H460	772	769	460	368	304	64	92	76	16
NHEK	1383	1378	755	604	450	154	151	112	39
SK.MEL.5	694	691	380	304	242	62	76	60	16
SK.N.DZ	762	759	422	337	205	132	85	52	33
SK.N.SH	2086	2077	1211	968	715	253	243	179	64
GM12878	2136	2128	1286	1028	788	240	258	198	60
K562	1197	1191	729	583	414	169	146	104	42
IMR.90	497	496	314	251	133	118	63	34	29

### Feature generation - Composition and correlation-based

For facilitating the training of machine learning (ML) models, we generated a large variety of features using different approaches. These features convert nucleotide sequences We used the in-house tool Nfeature (Mathur et al., 2021) for generating multiple composition and correlation features.

### Composition-based

Nucleotide composition-based features refer to quantitative representations of sequences that can be derived from the proportions and arrangements of nucleotides within these sequences. In this study, we have computed nucleic acid composition, distance distribution of nucleotides (DDN), nucleotide repeat index (NRI), pseudo composition and entropy of a sequence. The details for each of the features are provided in Table 2.

**TABLE 2:** Overview of the composition-based features generated using Nfeature.

Feature name	Type of descriptor	No. of descriptors
Nucleic acid Composition	Di-Nucleotide	16
Reverse complement K-Mer composition	Di-Nucleotide	10
Nucleotide Repeat Index	Mono-Nucleotide	4
Entropy	Sequence-level	1
Entropy	Nucleotide-level	4
Distance Distribution	Mono-Nucleotide	4
Pseudo Composition	Pseudo Di-nucleotide	19
Pseudo Composition	Pseudo Tri-nucleotide	65
	Total number of descriptors	123

### Correlation-based features

In this study, using Nfeature, we quantitatively assess the interdependent characteristics inherent in nucleotide sequences through the computation of correlation-based metrics. Correlation refers to the degree of relationship between distinct properties or features; an autocorrelation denotes the association of a feature with itself, whereas a cross-correlation indicates a linkage between two separate features. By employing these correlation-based descriptors, we effectively normalize the variable-length nucleotide sequences into uniform-length vectors, rendering them amenable to analysis via machine learning algorithms. These specific descriptors facilitate the identification and extraction of significant features predicated upon the nucleotide properties distributed throughout the sequence, enabling a more robust understanding of genetic information. A brief description of the features has been provided in Table 3.

**TABLE 3:** Overview of the correlation-based features generated using Nfeature.

Feature name	Type of descriptor	Number of descriptors
Cross Correlation	Trinucleotide Cross Correlation	264
Auto-Cross Correlation	Auto Dinucleotide - Cross Correlation	288
Auto-Cross Correlation	Auto Trinucleotide - Cross Correlation	288
Auto Correlation	Tri-Nucleotide	24
Auto Correlation	Normalized Moreau-Broto	24
Auto Correlation	Dinucleotide Moran	24
Auto Correlation	Dinucleotide Geary	24
Pseudo Correlation	Serial Correlation Pseudo Trinucleotide Composition	65
Pseudo Correlation	Serial Correlation Pseudo Dinucleotide Composition	17
Pseudo Correlation	Parallel Correlation Pseudo Trinucleotide Composition	65
Pseudo Correlation	Parallel Correlation Pseudo Dinucleotide Composition	17
	Total number of descriptors	1100

The total number of descriptors generated by using both composition and correlation-based features is 1223. Detailed explanation of the features and their biological implication have been provided in Supplementary Table S1. The properties used to calculate correlation-based features are provided in Supplementary Table S2.

### Embedding using DNABERT-2

DNABERT-2 is an adaptation of BERT (Bidirectional Encoder Representations from Transformers) designed specifically for DNA sequence analysis (Zhou et al., 2023). DNABERT-2 generates embeddings for DNA sequences that encapsulate not just the individual bases, but also their biological significance in terms of structure, function, and interactions. Moreover, the key advantage of DNABERT-2 embeddings lies in their ability to capture the complex dependencies within DNA sequences. We have made use of both aspects of DNABERT-2 - the pre-trained model to make predictions and the embeddings from the model to be used as features for downstream tasks. The pre-trained model was trained on the training dataset using the default parameters mentioned in their GitHub repository. Moreover, we have also generated embeddings from both the pre-trained as well as the fine-tuned models in order to use them as features for machine learning algorithms. The embeddings are derived from the hidden states of the model’s final output layer, using max pooling. The number of embeddings generated for each lncRNA using DNABERT-2 was 768.

### Five-fold cross validation

In order to estimate the performance of machine learning based models while training, we have deployed five-fold cross validation. In this method, the training dataset is split into five folds in a stratified manner and training is actually done over four folds and one fold is dedicated for validation. This process is iteratively performed for five times, by changing the fold that is used for validation and the rest of the folds being used for training. This generates an unbiased set of five performance metrics and the performance of the model is reported as the mean of these five sets.

### Feature selection

To optimize model performance and reduce computational complexity, we employed the Minimum Redundancy Maximum Relevance (mRMR) feature selection algorithm to identify the most informative features from our dataset. The mRMR algorithm selects features that exhibit the highest relevance to the target variable while minimizing redundancy among the features themselves, thereby enhancing the efficiency and predictive power of the models. For applying the mRMR algorithm, we combined all the features that were generated previously. Three different feature sets–Composition, Correlation and Embeddings, were used to generate a combined feature set comprising 2278 features. We evaluated the impact of feature selection by calculating and selecting subsets of 10, 50, 100, 500, 1000, 1500, and 2000 features. These subsets were subsequently used in downstream analyses to assess their influence on model performance metrics. Feature importance was also evaluated using simple correlation.

### Model development

In this study, three different approaches were followed for model development. The first approach involves the fine tuning of the DNABERT-2 using our training dataset and subsequently using the fine-tuned model to make predictions on the validation dataset. This method initially fine-tunes both the tokenizer and the pre-trained model according to our training dataset, and generates a fine-tuned tokenizer, and model. The fine-tuned model takes lncRNA sequences and generates the prediction using the tokenizer and model. In the next approach, we have implemented a hybrid approach, combining the components of large language models and machine learning algorithms. Instead of features, we have generated embeddings from a pre-trained as well as fine-tuned DNABERT-2 model. Embeddings from the DNABERT-2 model were then used to train machine learning models and subsequently evaluated them. The third approach involves composition and correlation-based features and using them to train machine learning models. The final model was developed using the.

### Model evaluation metrics

The binary classification performance of our fine-tuned model was evaluated using the following metrics: Sensitivity (SENS), Specificity (SPEC), Precision (PREC), Accuracy (ACC), Matthew’s Correlation Coefficient (MCC), F1-Score (F1) and Area Under the Receiver Operator Characteristic curve (AUC). The aforementioned metrics were calculated using the four different types of prediction outcomes: true positive (TP), false positive (FP), true negative (TN), and false negative (FN):

$$Sensitivity=\frac{TP}{TP+FN}$$

$$Specificity=\frac{TN}{TN+FP}$$

$$Precision=\frac{TP+TN}{TP+TN+FN+FP}$$

$$Accuracy=\frac{TP+TN}{TP+TN+FN+FP}$$

$$MCC=\frac{\left(TP\times TN\;\right)-\left(FP\times FN\right)}{\sqrt{\left(TP+FP\right)\times \left(TP+FN\right)\times \left(TN+FP\right)\times \left(TN+FN\right)}}$$

$$F1-score=\frac{TP}{TP+\left(0.5\times \left(FN+FP\right)\right)}$$

The evaluation of our binary classification model using various metrics provides critical insights into its performance. Sensitivity (SENS) measures the model’s ability to identify positive instances, while Specificity (SPEC) assesses its accuracy in recognizing negative instances. Precision (PREC) reflects the accuracy of positive predictions, and Accuracy (ACC) offers an overall measure of correctness, though it may be misleading in imbalanced datasets. Matthew’s Correlation Coefficient (MCC) provides a balanced view by considering all prediction outcomes, with values close to 1 indicating strong predictive capability. The F1-Score (F1) combines Precision and Sensitivity into a single metric, ideal for balancing the trade-off between false positives and negatives. Finally, the Area Under the Curve (AUC) evaluates the model’s ability to distinguish between classes across different thresholds, with higher values indicating better performance. Together, these metrics enable a comprehensive evaluation of the model, guiding necessary improvements and refinements.

## Results

**FIGURE 4:**
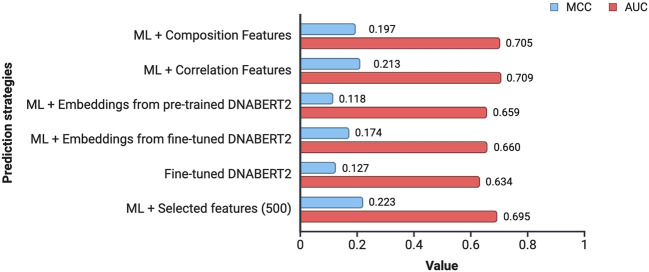
Overview of the performance achieved by different prediction strategies. The values indicate the average MCC and AUC across all the 15 cell-lines for a prediction strategy.

In this study, an attempt was made to design a model that will be able to classify the subcellular location of lncRNA into cytoplasm or nucleus. To achieve this, we tried out multiple approaches. Figure 4 provides an overview of the performance of the various approaches tried in this study.

### Functional enrichment analysis

The GO and KEGG enrichment analysis, conducted using RNAenrich (Zhang et al., 2023), reveals distinct functional roles for cytoplasmic versus nuclear-localizing lncRNAs. Among the significantly enriched GO terms (adjusted p-value <0.05), 2,511 were shared, while 397 were unique to cytoplasmic lncRNAs (positive class) and 254 to nuclear lncRNAs (negative class). Cytoplasmic lncRNAs were enriched for biological processes such as “response to interferon-beta” (GO:0035456), “positive regulation of apoptotic process” (GO:0043065), and “RNA splicing” (GO:0008380), indicating roles in immune signaling, post-transcriptional regulation, and cellular stress responses. Correspondingly, KEGG pathway enrichment identified associations with Ferroptosis (hsa04216) and Autophagy (hsa04140), further highlighting their involvement in cytoplasmic stress and degradation pathways. In contrast, nuclear-localized lncRNAs were enriched for GO terms such as “eukaryotic 48S preinitiation complex” (GO:0033290), “regulation of transcription of nucleolar large rRNA by RNA polymerase I” (GO:1901836), and “MLL1/2 complex” (GO:0044665), reflecting their roles in transcriptional regulation, chromatin remodeling, and nucleolar function. KEGG analysis further linked nuclear lncRNAs to Sterol Biosynthesis (hsa00100) and Nucleotide Excision Repair (hsa03420), pointing to nuclear functions in genome maintenance and metabolic regulation. Together, these enrichments underscore the compartment-specific biological functions of lncRNAs, shaped by their cellular localization.

### Feature importance

To identify features associated with subcellular localization labels (cytoplasm or nucleus), we computed the correlation of each feature with the corresponding CNRCI values. A positive correlation indicates that an increase in the feature value favors cytoplasmic localization, whereas a negative correlation suggests a preference for nuclear localization. This analysis elucidates which features predominantly influence localization to either compartment. The top 10 genes that were highly correlated with the CNRCI values are provided in Table 4, 5 for composition-based and correlation-based features, respectively. A more detailed version of this table is provided in Supplementary Table S3, 4. It can be observed that Cytosine-based k-mers are more prevalent in the positively correlated features (supporting cytoplasm localization) whereas Thymine is predominantly found in negatively correlated features (supporting nucleus localization).

**TABLE 4:** Composition based-features having the highest correlation with the CNRCI values for 15 cell-lines.

	Top 5 positively correlated features					Top 5 negatively correlated features				
SK.N.DZ	CDK_CGA	CDK_TCG	CDK_AAC	CDK_ACG	CDK_CG	CDK_TGG	CDK_TG	CDK_CTG	CDK_GGG	CDK_GGT
HeLa.S3	CDK_TAC	CDK_AA	CDK_AAT	CDK_A	CDK_TA	CDK_GG	CDK_G	CDK_GGG	CDK_GGC	CDK_CTG
HUVEC	CDK_CG	CDK_GCG	CDK_CGG	CDK_CGA	CDK_CGC	CDK_TG	CDK_CAT	CDK_T	CDK_TGA	CDK_ATG
NHEK	CDK_GCG	CDK_CGC	CDK_CCG	CDK_CG	CDK_CGG	CDK_TG	CDK_TGT	CDK_GTG	CDK_GT	CDK_TCT
GM12878	CDK_CGA	CDK_ACG	CDK_CG	CDK_GCG	CDK_TCG	CDK_CAT	CDK_TCA	CDK_TAT	CDK_AT	CDK_TTC
IMR.90	CDK_CGA	CDK_GCG	CDK_CG	CDK_CGG	CDK_CGC	CDK_TG	CDK_TGG	CDK_CTG	CDK_CCT	CDK_CT
A549	CDK_CGA	CDK_AAA	CDK_AA	CDK_GCG	CDK_GAA	CDK_CTG	CDK_CA	CDK_CAG	CDK_TG	CDK_CCA
MCF.7	CDK_CGA	CDK_CG	CDK_GCG	CDK_CGC	CDK_CGG	CDK_TG	CDK_ATG	CDK_CAT	CDK_TGT	CDK_T
NCI.H460	CDK_AAC	CDK_CGA	CDK_CAA	CDK_ACG	CDK_CGT	CDK_TGG	CDK_TG	CDK_GGG	CDK_GTG	CDK_GGT
SK.MEL.5	CDK_CGC	CDK_CG	CDK_CCG	CDK_ACG	CDK_CGA	CDK_AGT	CDK_GAT	CDK_TGA	CDK_TG	CDK_TTG
H1.hESC	CDK_TA	CDK_TTA	CDK_TAA	CDK_AAT	CDK_AA	CDK_CTG	CDK_CAG	CDK_C	CDK_CCA	CDK_CC
HT1080	CDK_CGA	CDK_CG	CDK_GCG	CDK_ACG	CDK_CGC	CDK_TG	CDK_T	CDK_ATG	CDK_TGT	CDK_TTT
K562	CDK_CGA	CDK_CG	CDK_GCG	CDK_ACG	CDK_CGC	CDK_CAG	CDK_AG	CDK_CTG	CDK_TGG	CDK_CA
HepG2	CDK_CGA	CDK_CG	CDK_TCG	CDK_GCG	CDK_CGC	CDK_TG	CDK_CT	CDK_CTG	CDK_CA	CDK_TGT
SK.N.SH	CDK_AA	CDK_AAC	CDK_AAA	CDK_TAA	CDK_CAA	CDK_CTG	CDK_TG	CDK_TGG	CDK_CAG	CDK_CCA

**TABLE 5:** Correlation-based features having the highest correlation with the CNRCI values for 15 cell-line.

	Top 5 positively correlated features					Top 5 negatively correlated features				
SK.N.DZ	TCC_p3_p4_lag1	TACC_p3_p4_lag1	TCC_p3_p11_lag1	TACC_p3_p11_lag1	TCC_p2_p3_lag2	DACC_p1_p4_lag1	TCC_p3_p12_lag1	TACC_p3_p12_lag1	TCC_p12_p3_lag1	TACC_p12_p3_lag1
HeLa.S3	DACC_p5_p3_lag1	PC_PTNC_TAG	SC_PTNC_TAG	PKNC_TAG	TAC_p1_lag1	TCC_p1_p8_lag1	TACC_p1_p8_lag1	TCC_p8_p1_lag1	TACC_p8_p1_lag1	TCC_p2_p8_lag1
HUVEC	DACC_p7_lag1	DACC_p9_p8_lag1	DACC_p1_p7_lag1	DACC_p4_p2_lag1	DACC_p4_p8_lag1	DACC_p9_p7_lag1	DACC_p1_p8_lag1	DACC_p4_p7_lag1	DACC_p7_p2_lag1	DACC_p7_p4_lag1
NHEK	DACC_p4_p2_lag1	DACC_p10_p2_lag1	DACC_p6_p2_lag1	DACC_p7_p12_lag1	DACC_p9_p4_lag1	DACC_p7_p2_lag1	DACC_p4_p12_lag1	DACC_p6_p12_lag1	DACC_p9_p7_lag1	PC_PDNC_TT
GM12878	TCC_p9_p3_lag1	TCC_p10_p3_lag1	TACC_p9_p3_lag1	TACC_p10_p3_lag1	TCC_p3_p9_lag1	DACC_p9_p7_lag1	DACC_p4_p7_lag1	DACC_p7_p4_lag1	DACC_p7_p6_lag1	DACC_p6_p7_lag1
IMR.90	DACC_p4_p2_lag1	DACC_p6_p2_lag1	DACC_p1_p7_lag1	DACC_p10_p2_lag1	MAC_p2_lag1	DACC_p6_p12_lag1	DACC_p7_p2_lag1	DACC_p9_p7_lag1	DACC_p1_p2_lag1	DACC_p4_p12_lag1
A549	DACC_p1_lag1	DACC_p4_lag1	DACC_p8_p9_lag2	TAC_p3_lag1	TACC_p3_lag1	DACC_p9_p1_lag1	DACC_p4_p1_lag1	DACC_p8_p1_lag2	DACC_p1_p4_lag1	DACC_p7_p9_lag2
MCF.7	DACC_p7_lag1	DACC_p1_p7_lag1	DACC_p9_p8_lag1	DACC_p4_p2_lag1	DACC_p4_p8_lag1	DACC_p1_p8_lag1	DACC_p9_p7_lag1	DACC_p4_p7_lag1	DACC_p7_p2_lag1	DACC_p7_p4_lag1
NCI.H460	DACC_p9_p4_lag1	DACC_p1_p7_lag1	DACC_p9_p6_lag1	DACC_p7_p12_lag1	DACC_p4_p6_lag1	DACC_p1_p10_lag1	DACC_p1_p6_lag1	DACC_p1_p4_lag1	DACC_p4_p12_lag1	DACC_p10_p12_lag1
SK.MEL.5	DACC_p9_p8_lag1	DACC_p4_p2_lag1	DACC_p9_p2_lag1	DACC_p10_p2_lag1	SC_PTNC_CGG	TCC_p3_p7_lag1	TACC_p3_p7_lag1	DACC_p9_p7_lag1	TCC_p7_p3_lag1	TACC_p7_p3_lag1
H1.hESC	NMBAC_p1_lag1	DACC_p3_p11_lag1	DACC_p1_lag1	DACC_p3_p5_lag1	PDNC_TC	PKNC_CTT	PKNC_CAT	SC_PTNC_CTT	PC_PTNC_CTT	SC_PTNC_CAT
HT1080	DACC_p4_p2_lag1	DACC_p1_p7_lag1	DACC_p10_p2_lag1	DACC_p7_lag1	DACC_p6_p2_lag1	DACC_p9_p7_lag1	DACC_p1_p8_lag1	DACC_p7_p2_lag1	DACC_p1_p2_lag1	DACC_p4_p7_lag1
K562	TCC_p9_p3_lag1	TCC_p10_p3_lag1	TACC_p9_p3_lag1	TACC_p10_p3_lag1	TCC_p3_p9_lag1	DACC_p1_p8_lag2	DACC_p1_p4_lag1	DACC_p1_p10_lag2	DACC_p4_p1_lag2	DACC_p9_p7_lag2
HepG2	DACC_p7_p1_lag1	TAC_p3_lag1	TACC_p3_lag1	DACC_p4_lag1	DACC_p1_p7_lag1	DACC_p4_p7_lag1	DACC_p7_p4_lag1	DACC_p7_p6_lag1	DACC_p6_p7_lag1	DACC_p9_p7_lag1
SK.N.SH	DACC_p1_lag1	DACC_p1_p7_lag1	DACC_p9_p4_lag1	DACC_p8_p9_lag2	TCC_p11_p3_lag1	DACC_p1_p4_lag1	DACC_p9_p1_lag1	DACC_p4_p1_lag1	DACC_p1_p6_lag1	TCC_p12_p3_lag1

**FIGURE 5:**
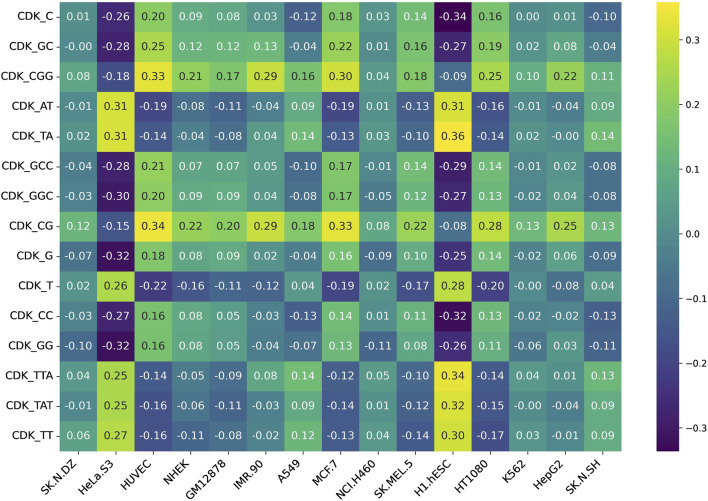
Heatmap depicting the composition-based features that demonstrate the highest variation in their correlation with the CNRCI values within 15 cell-lines.

**FIGURE 6:**
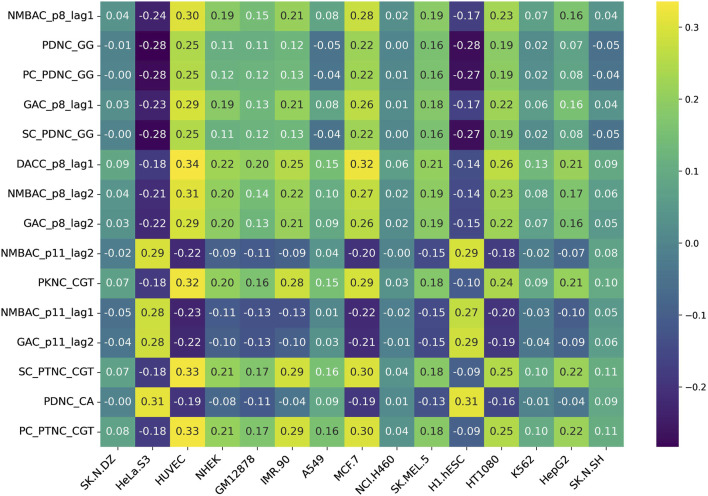
Heatmap depicting the correlation-based features that demonstrate the highest variation in their correlation with the CNRCI values within 15 cell-lines.

Additionally, we assessed feature variability across cell lines by calculating the difference between the maximum and minimum correlation values observed for each feature across all 15 cell lines. This approach highlights features exhibiting the most pronounced inter-cell-line variation, providing insight into their potential biological or experimental variability. Figures 5, 6 represent heatmaps depicting the highly variable genes and their correlation with the CNRCI values for composition and correlation-based features, respectively. The complete information for the variable genes has been provided in Supplementary Table S5, 6.

### Model based on composition and correlation features

Composition and correlation features generated from Nfeature were used to train multiple ML models. We have computed the performance of nine composition-based features and thirteen correlation-based features. We implemented all the combinations of feature and ML model to identify which feature-ML model combination performs the best. The composition features combined with classical ML methods were able to achieve an average AUC of 0.7049 and a MCC of 0.1965, across the 15 cell-lines. Similarly, with correlation-based features and ML methods, the best performance achieved was an average AUC of 0.7089 and a MCC of 0.2133. Performance of the best performing model using both composition and correlation-based features are provided for all the 15 cell-lines in Table 6, 7 respectively. The detailed performance for all the models used in this analysis have been provided in Supplementary Table S7, 8. The model parameters are provided in Supplementary Table S12.

**TABLE 6:** Performance of the best ML model for each cell-line on the validation dataset using composition features.

Cell-lines	Feature used	ML model used	Sensitivity	Specificity	Precision	Accuracy	MCC	F1-score	AUC
A549	RDK	RandomForestClassifier	0.600	0.825	0.692	0.736	0.438	0.643	0.761
H1.hESC	CDK	RandomForestClassifier	0.500	0.785	0.607	0.671	0.297	0.548	0.708
HeLa.S3	PKNC	GaussianNB	0.826	0.593	0.284	0.631	0.310	0.422	0.779
HepG2	RDK	GaussianNB	0.429	0.847	0.511	0.733	0.292	0.466	0.718
HT1080	CDK	MLPClassifier	0.517	0.769	0.633	0.659	0.296	0.569	0.728
HUVEC	PKNC	SVC	0.032	0.964	0.250	0.706	−0.011	0.056	0.756
MCF.7	CDK	GaussianNB	0.452	0.809	0.437	0.721	0.259	0.444	0.724
NCI.H460	PKNC	SVC	0.000	1.000	0.000	0.826	0.000	0.000	0.723
NHEK	CDK	SVC	0.000	1.000	0.000	0.742	0.000	0.000	0.643
SK.MEL.5	CDK	XGBClassifier	0.063	0.950	0.250	0.763	0.023	0.100	0.676
SK.N.DZ	CDK	MLPClassifier	0.545	0.692	0.529	0.635	0.237	0.537	0.679
SK.N.SH	PDNC	QuadraticDiscriminantAnalysis	0.547	0.732	0.422	0.683	0.259	0.476	0.703
GM12878	PKNC	GradientBoostingClassifier	0.133	0.939	0.400	0.752	0.115	0.200	0.687
K562	ALL_COMP	DecisionTreeClassifier	0.452	0.788	0.463	0.692	0.243	0.458	0.620
IMR.90	PKNC	AdaBoostClassifier	0.448	0.735	0.591	0.603	0.192	0.510	0.668
Average			0.370	0.829	0.405	0.704	0.197	0.362	0.705

**TABLE 7:** Performance of the best ML model for each cell-line on the validation dataset using correlation features.

Cell-lines	Feature used	ML model used	Sensitivity	Specificity	Precision	Accuracy	MCC	F1-score	AUC
A549	PC_PDNC	GradientBoostingClassifier	0.567	0.803	0.654	0.709	0.381	0.607	0.757
H1.hESC	PDNC	RandomForestClassifier	0.495	0.811	0.635	0.685	0.324	0.556	0.720
HeLa.S3	PKNC	GaussianNB	0.783	0.585	0.269	0.617	0.272	0.400	0.779
HepG2	MAC	GaussianNB	0.679	0.567	0.369	0.597	0.218	0.478	0.715
HT1080	SC_PTNC	GaussianProcessClassifier	0.583	0.769	0.660	0.688	0.359	0.619	0.738
HUVEC	PDNC	AdaBoostClassifier	0.302	0.897	0.528	0.732	0.243	0.384	0.757
MCF.7	SC_PDNC	SVC	0.000	1.000	0.000	0.754	0.000	0.000	0.753
NCI.H460	PDNC	SVC	0.000	1.000	0.000	0.826	0.000	0.000	0.683
NHEK	PKNC	AdaBoostClassifier	0.231	0.866	0.375	0.702	0.116	0.286	0.635
SK.MEL.5	PDNC	GradientBoostingClassifier	0.063	0.933	0.200	0.750	−0.007	0.095	0.654
SK.N.DZ	TAC	SVC	0.424	0.865	0.667	0.694	0.327	0.519	0.739
SK.N.SH	PC_PTNC	GaussianNB	0.750	0.531	0.364	0.588	0.248	0.490	0.689
GM12878	PC_PDNC	XGBClassifier	0.350	0.899	0.512	0.771	0.288	0.416	0.715
K562	DACC	AdaBoostClassifier	0.405	0.808	0.459	0.692	0.221	0.430	0.642
IMR.90	PC_PTNC	AdaBoostClassifier	0.621	0.588	0.563	0.603	0.208	0.590	0.655
Average			0.417	0.795	0.417	0.694	0.213	0.391	0.709

### Models based on embeddings from DNABERT-2

Embeddings from large language models are known to encapsulate not just the individual bases, but also their biological significance in terms of structure, function, and interactions. In this approach, we generated high level representations of lncRNA sequences using both the pre-trained as well as the fine-tuned models. These embeddings were used to train ML models and the models were evaluated on the validation dataset. In the case of pre-trained embeddings, the model achieved an average AUC of 0.6586 and an average MCC of 0.1182. When fine-tuned embeddings were used as features, the performance of the model dropped marginally, achieving an average AUC of 0.6604 and an average MCC of 0.1740. Detailed results for the performance of ML models on the validation dataset using pre-trained as well as fine-tuned embeddings as features are provided in Table 8, 9 respectively. The detailed performance for all the models has been reported in Supplementary Table S9, 10.

**TABLE 8:** Performance of the best ML model for each cell-line on the validation dataset using embeddings from pre-trained DNABERT-2 model.

Cell-lines	ML model used	Sensitivity	Specificity	Precision	Accuracy	MCC	F1-score	AUC
A549	SVC linear	0.500	0.759	0.577	0.656	0.267	0.536	0.661
H1.hESC	MLP Classifier	0.588	0.638	0.519	0.618	0.223	0.552	0.666
HeLa.S3	XGBoost Classifier	0.087	0.992	0.667	0.844	0.201	0.154	0.699
HepG2	MLP Classifier	0.000	1.000	0.000	0.728	0.000	0.000	0.706
HT1080	MLP Classifier	0.817	0.513	0.563	0.645	0.338	0.667	0.735
HUVEC	Random Forest Classifier	0.032	0.982	0.400	0.719	0.041	0.059	0.690
MCF.7	MLP Classifier	0.012	0.984	0.200	0.745	−0.013	0.022	0.657
NCI.H460	Gradient Boosting Classifier	0.063	1.000	1.000	0.837	0.228	0.118	0.599
NHEK	Gaussian Naive Bayes Classifier	0.538	0.705	0.389	0.662	0.223	0.452	0.660
SK.MEL.5	KNN	0.063	0.967	0.333	0.776	0.061	0.105	0.638
SK.N.DZ	Gradient Boosting Classifier	0.364	0.808	0.545	0.635	0.191	0.436	0.678
SK.N.SH	MLP Classifier	0.469	0.709	0.366	0.646	0.166	0.411	0.618
GM12878	Logistic Regression	0.117	0.955	0.438	0.760	0.125	0.184	0.655
K562	Logistic Regression	0.071	0.923	0.273	0.678	−0.009	0.113	0.609
IMR.90	Random Forest Classifier	0.345	0.824	0.625	0.603	0.193	0.444	0.699
Average		0.271	0.851	0.460	0.704	0.149	0.284	0.665

**TABLE 9:** Performance of the best ML model for each cell-line on the validation dataset using embeddings from fine-tuned DNABERT-2 model.

Cell-lines	ML model used	Sensitivity	Specificity	Precision	Accuracy	MCC	F1-score	AUC
A549	SVC_linear	0.489	0.708	0.524	0.621	0.200	0.506	0.660
H1.hESC	SVC_linear	0.422	0.801	0.585	0.650	0.241	0.490	0.687
HeLa.S3	MLP Classifier	0.000	0.992	0.000	0.830	−0.037	0.000	0.732
HepG2	Gaussian Naive Bayes Classifier	0.571	0.767	0.478	0.714	0.321	0.520	0.708
HT1080	SVC_linear	0.450	0.756	0.587	0.623	0.217	0.509	0.700
HUVEC	MLP Classifier	0.206	0.903	0.448	0.711	0.147	0.283	0.726
MCF.7	Gaussian Naive Bayes Classifier	0.512	0.739	0.391	0.683	0.232	0.443	0.700
NCI.H460	XGBoost Classifier	0.000	0.987	0.000	0.815	−0.048	0.000	0.535
NHEK	AdaBoost Classifier	0.359	0.821	0.412	0.702	0.189	0.384	0.600
SK.MEL.5	SVC_radial	0.000	1.000	0.000	0.789	0.000	0.000	0.539
SK.N.DZ	Gradient Boosting Classifier	0.515	0.692	0.515	0.624	0.207	0.515	0.717
SK.N.SH	AdaBoost Classifier	0.156	0.866	0.294	0.679	0.028	0.204	0.587
GM12878	Gradient Boosting Classifier	0.183	0.939	0.478	0.764	0.182	0.265	0.683
K562	Gradient Boosting Classifier	0.143	0.894	0.353	0.678	0.052	0.203	0.590
IMR.90	Gradient Boosting Classifier	0.552	0.706	0.615	0.635	0.261	0.582	0.671
Average		0.304	0.838	0.379	0.701	0.146	0.327	0.656

### Fine-tuned DNABERT-2 model

In this approach, we used our training dataset to fine-tune the model and generate a fine-tuned tokenizer and model. Using this tokenizer and model, we generate high level representations of our lncRNA sequences and these representations are used by the model to generate predictions. The fine-tuned DNABERT2 model could not be evaluated while training as we were not able to implement five-fold cross validation. The fine-tuned model was evaluated on the validation dataset and performance metrics for the same are reported in Table 10. The detailed performance for all the models has been provided in Supplementary Table S11.

**TABLE 10:** Performance of fine-tuned DNABERT-2 model on the validation dataset.

Cell-lines	Sensitivity	Specificity	Precision	Accuracy	MCC	F1-score	AUC
A549	0.500	0.861	0.703	0.718	0.393	0.584	0.762
H1.hESC	0.520	0.713	0.546	0.636	0.235	0.533	0.644
HeLa.S3	0.000	1.000	0.000	0.837	0.000	0.000	0.527
HepG2	0.000	1.000	0.000	0.728	0.000	0.000	0.698
HT1080	0.567	0.731	0.618	0.659	0.301	0.591	0.676
HUVEC	0.159	0.976	0.714	0.750	0.251	0.260	0.710
MCF.7	0.000	1.000	0.000	0.754	0.000	0.000	0.562
NCI.H460	0.000	1.000	0.000	0.826	0.000	0.000	0.576
NHEK	0.000	1.000	0.000	0.742	0.000	0.000	0.564
SK.MEL.5	0.000	1.000	0.000	0.789	0.000	0.000	0.468
SK.N.DZ	0.515	0.885	0.739	0.741	0.439	0.607	0.791
SK.N.SH	0.000	1.000	0.000	0.737	0.000	0.000	0.589
GM12878	0.000	1.000	0.000	0.767	0.000	0.000	0.718
K562	0.190	0.885	0.400	0.685	0.099	0.258	0.570
IMR.90	0.655	0.529	0.543	0.587	0.185	0.594	0.649
Average	0.207	0.905	0.284	0.730	0.127	0.228	0.634

### Model based on features selected by mRMR algorithm

In order to identify the best set of features from the combined feature set of 2278 features (composition, correlation, and embeddings), mRMR algorithm was used. Seven different feature sets were created based on the top ‘k’ features selected by mRMR. Performance was evaluated for seven different sets of features for each cell-line using 12 different ML classifiers. Table 11 reports the AUC for the best model for each combination and the last row shows the average for each feature set. The best AUC value was reported when top 500 genes selected by mRMR was used for training.

**TABLE 11:** AUC values of the best ML model for each cell-line on the validation dataset for the feature sets generated by mRMR algorithm.

Cell lines	Top 10	Top 50	Top 100	Top 500	Top 1000	Top 1500	Top 2000
A549	0.756	0.75	0.749	0.741	0.739	0.738	0.738
GM12878	0.725	0.706	0.706	0.727	0.742	0.734	0.719
H1.hESC	0.677	0.687	0.694	0.71	0.712	0.716	0.717
HT1080	0.71	0.73	0.728	0.731	0.745	0.749	0.742
HUVEC	0.733	0.744	0.762	0.771	0.76	0.748	0.764
HeLa.S3	0.669	0.728	0.756	0.734	0.718	0.776	0.779
HepG2	0.736	0.722	0.725	0.72	0.691	0.673	0.685
IMR.90	0.725	0.651	0.647	0.621	0.636	0.602	0.663
K562	0.615	0.665	0.645	0.628	0.585	0.581	0.586
MCF.7	0.706	0.728	0.73	0.701	0.715	0.721	0.711
NCI.H460	0.535	0.514	0.533	0.638	0.553	0.593	0.568
NHEK	0.64	0.634	0.626	0.609	0.603	0.632	0.65
SK.MEL.5	0.672	0.652	0.552	0.725	0.597	0.619	0.575
SK.N.DZ	0.671	0.664	0.675	0.704	0.705	0.715	0.707
SK.N.SH	0.683	0.684	0.685	0.669	0.683	0.684	0.682
Average	0.683	0.684	0.681	0.695	0.679	0.685	0.686

### Performance comparison of CytoLNCpred and existing state-of-the-art classifiers

To further illustrate the efficacy of our method, we conduct a comparative analysis with other cutting-edge classifiers. Specifically, we evaluate existing predictors, namely, lncLocator 2.0 and TACOS, which employ predictive algorithms to predict subcellular location of lncRNAs in different cell-lines.

Among these predictors, lncLocator 2.0 relies on the word embeddings and a MultiLayer Perceptron Regressor to predict CNRCI values. The predicted CNRCI values were then converted to labels using a fixed threshold value. The second predictor, TACOS, generated a variety of feature encodings using composition and physicochemical properties and tree-based algorithms were deployed to make the predictions. It is important to note that TACOS has been trained on 10 out of the 15 cell-lines. For a fair performance comparison, we leverage the performance metrics of evaluated on the validation dataset. Table 12 summarizes the evaluation of CytoLNCpred and other existing tools based on AUROC.

**TABLE 12:** Comparing the performance of our method and other existing classifiers using our validation dataset based on AUROC for all cell-lines.

	lncLocator 2.0	TACOS	CytoLncPred – Composition features	CytoLncPred – Correlation features
A549	0.592	0.741	0.761	0.757
H1.hESC	0.649	0.752	0.708	0.720
HeLa.S3	0.492	0.721	0.779	0.779
HepG2	0.487	0.712	0.718	0.715
HT1080	0.597	0.727	0.728	0.738
HUVEC	0.500	0.721	0.756	0.757
MCF.7	0.530	-	0.724	0.753
NCI.H460	0.500	-	0.723	0.683
NHEK	0.519	0.623	0.643	0.635
SK.MEL.5	0.500	0.567	0.676	0.654
SK.N.DZ	0.500	-	0.679	0.739
SK.N.SH	0.508	0.633	0.703	0.689
GM12878	0.500	0.568	0.687	0.715
K562	0.500	-	0.620	0.642
IMR.90	0.471	-	0.668	0.655
Average	0.523	0.676	0.705	0.709

### mRNA localization prediction accuracy using CytoLNCpred

**FIGURE 7:**
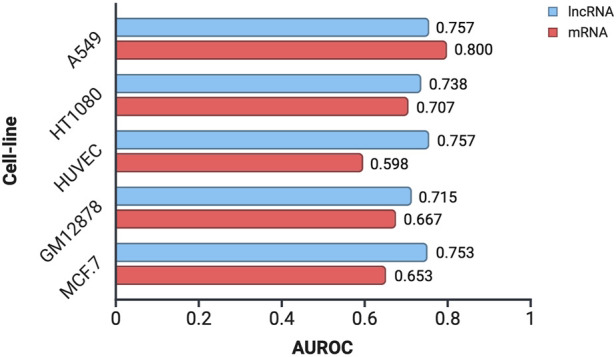
Performance evaluation of CytoLNCpred in predicting the subcellular localization of mRNA and lncRNA sequences in various cell lines, as measured by the Area Under the Receiver Operating Characteristic curve (AUROC).

To assess the applicability of CytoLNCpred, a tool originally developed for lncRNA localization prediction, to mRNA sequences, we utilized mRNA data obtained from the lncAtlas database. These mRNA sequences were subjected to prediction using the standalone version of CytoLNCpred, and its performance was evaluated using the Area Under the Receiver Operating Characteristic curve (AUROC). The AUROC values obtained for mRNA localization prediction across different cell lines, alongside the corresponding performance for lncRNA prediction, are presented in Figure 7.

The results indicate that CytoLNCpred exhibits a varying degree of accuracy in predicting the subcellular localization of mRNA sequences across the tested cell lines. As illustrated in Figure 7, the predictive performance for mRNA localization differs depending on the cellular context. Notably, the highest prediction accuracy for mRNA was observed in the A549 cell line (AUROC = 0.800), suggesting a strong potential for the tool in this specific context. While the performance varied across different cell lines, with HUVEC showing the lowest AUROC (0.598), the overall results suggest that features learned by CytoLNCpred for lncRNA localization can also provide some discriminatory power for mRNA localization. Interestingly, in the A549 cell line, the prediction accuracy for mRNA even surpassed that observed for lncRNAs. However, in other cell lines like HUVEC and MCF-7, the performance on mRNA was notably lower compared to lncRNAs.

In order to further validate the model accuracy for mRNAs, 10 random cytoplasmic mRNAs were obtained from NCBI gene database. These mRNAs were then predicted using CytoLNCpred for the A549 cell-line. The model was able to predict only 2 mRNAs correctly, among the 10 cytoplasmic RNA and the detailed results are provided in Supplementary Table S13. This variability in performance across cell types highlights the potential influence of cell-specific factors on mRNA localization and suggests that further refinement or specialized models might be beneficial for broader applicability to mRNA.

## Discussion

In recent years, researchers have recognized that the subcellular localization of lncRNAs plays a pivotal role in understanding their function. Unlike protein-coding genes, lncRNAs do not encode proteins directly. Instead, they exert their effects through diverse mechanisms, including interactions with chromatin, RNA molecules, and proteins. The precise localization of lncRNAs within the cell provides crucial information about their regulatory roles.

In our analysis LINC00852 showed a marked cell-line–specific shift–predominantly nuclear (negative localization score) in most cell types, but strongly cytoplasmic in the NCI-H460 lung carcinoma line. This mirrors literature reports that LINC00852 can be cytoplasmically enriched in lung carcinoma cases. In lung carcinoma cell-lines (A549 and SPCA-1), LINC00852 was found mainly in the cytoplasm (qRT-PCR assay) (Liu et al., 2018). It binds with the S100A9 protein in the cytoplasm, activating the MAPK pathway and plays a positive role in the progression and metastasis of lung adenocarcinoma cells. By contrast, other studies have observed LINC00852 in the nucleus in some tumors. In osteosarcoma cell-lines (like 143B and MG-63), it is observed that LINC00852 acts as a transcription factor and increase the expression of AXL gene (Li et al., 2020). Such context dependence could reflect tissue-specific expression of RNA-binding factors or isoforms that govern nuclear export. The fact that LINC00852 is cytoplasmic in some cancer lines but nuclear in others suggests it may switch roles-in cytosolic form it may acts as a post-transcriptional regulator (e.g., miRNA sponge), whereas nuclear retention may imply transcriptional or chromatin-related roles.

SNHG3 in our data is mostly cytoplasmic in H1.hESC, HepG2 (liver carcinoma) and GM12878 (lymphoblast) cells, but nuclear in cell-lines like MCF-7 and NHEK. This pattern aligns with experimental studies. In colorectal cancer cell lines (e.g., SW480, LoVo), SNHG3 was found to localize predominantly in the cytoplasm (comparable to GAPDH) (Huang et al., 2017). There it acts as a competing endogenous RNA (ceRNA), sponging miRNAs (e.g., miR-182-5p) to upregulate oncogenic targets like c-Myc. The high cytoplasmic SNHG3 expression in stem-cell like and proliferative cell-lines (H1.hESC, HepG2) from our dataset suggests a similar ceRNA role, whereas its nuclear enrichment in more differentiated/epithelial cells may reflect downregulation of this pathway in those contexts. In general, SNHG-family lncRNAs are known to influence cancer cell growth and often operate via cytoplasmic post-transcriptional mechanisms, consistent with SNHG3’s localization and function in promoting malignancy.

Subcellular localization of lncRNA gains prominence in recent times due to their role in gene regulation within the cell. A large number of aptamer and ASO based drugs are being developed using RNA nanotechnology. In recent years, the convergence of nanotechnology and long non-coding RNAs (lncRNAs) has yielded exciting developments in drug development. Nanoparticles, such as liposomes and exosomes, are being harnessed for targeted delivery of lncRNA-based therapeutics to cancer cells. Additionally, CRISPR-Cas9 technology, delivered via nanoparticles, enables precise gene editing by modulating lncRNA expression. Computational models and deep learning approaches are aiding our understanding of lncRNA-mediated mechanisms. Overall, this interdisciplinary field holds immense promise for personalized medicine, improved therapies, and better patient outcomes.

Predicting lncRNA subcellular localization using tools like CytoLNCpred offers significant potential for guiding the development of RNA-based therapeutics and CRISPR strategies. Since antisense oligonucleotides (ASOs) are generally more effective against nuclear lncRNAs (Zong et al., 2015) and small interfering RNAs (siRNAs) excel against cytoplasmic targets (Lennox and Behlke, 2016), a CytoLNCpred prediction indicating cytoplasmic enrichment would favor siRNA development, while a predicted nuclear localization would suggest ASOs as the primary choice. Similarly, this prediction informs CRISPR approaches: targeting nuclear-acting lncRNAs might be best achieved by disrupting transcription or key regulatory elements using CRISPRi or Cas9 (Rosenlund et al., 2021), whereas lncRNAs predicted to function in the cytoplasm could be more effectively targeted by degrading the transcript directly using RNA-targeting CRISPR-Cas13 (Xu et al., 2020), potentially guiding crRNA expression strategies (e.g., using a U1 promoter for cytoplasmic crRNA localization). Thus, localization prediction aids in the rational selection of therapeutic modalities and CRISPR targeting strategies based on the likely site of lncRNA function.

In recent times large language models are considered as SOTA methods and apart from the classical composition and correlation feature-based model, we also implemented DNABERT-2 for our classification problem. The DNABERT-2 model has been trained on the genomes of a wide variety of species and is computationally very efficient. DNABERT-2 uses Byte Pair Encoding to generate tokens which is known to perform better than k-mer tokenization. So, in order to fully exploit the DNABERT-2 model, we generated embeddings from both pre-trained and fine-tuned models. These embeddings when combined with ML methods were able to predict subcellular localization very well but poorer than a fine-tuned DNABERT-2 model.

In our study, we compared the performance of DNABERT-2 with traditional composition and correlation-based features for classifying subcellular localization of lncRNAs. While DNABERT-2, a pre-trained language model, showed promising results, but we found that traditional machine learning models trained on carefully crafted composition and correlation features consistently outperformed DNABERT-2. This suggests that for this specific task, the carefully engineered features capture the relevant biological information more effectively than the general-purpose representations learned by DNABERT-2. Specifically, it was observed that the correlation-based features achieve a higher average AUC than all other approaches. However, these approaches failed when we used RNA sequences greater than 10,000 base pairs. In order to reduce the variability in nucleotide length, the sequence length was limited to 10,000 base pairs.

The lncAtlas database, while a valuable resource for lncRNA subcellular localization, has several significant limitations including its restriction to GENCODE-annotated lncRNAs and a limited set of 15 human cell lines, with detailed sub-compartment data available only for the K562 cell line. The database relies on RNA-seq data and the Relative Concentration Index (RCI), which provides relative abundance rather than absolute counts. Furthermore, lncAtlas has not been updated since 2017, making it less comprehensive and potentially outdated.

To address these limitations, future research should focus on developing techniques to improve the interpretability of DNABERT-2’s predictions. This could involve methods such as attention visualization or feature importance analysis. Furthermore, expanding the diversity of training data is essential to enhance the model’s generalizability across different biological contexts. By incorporating data from a wider range of organisms and conditions, subcellular localization prediction could become a more versatile and reliable tool for genomic analysis. In our case, correlation-based features with machine learning algorithms outperformed all other approaches. Moreover, improved machine learning algorithms are needed to be developed that can account for large variability in nucleotide lengths.

## Conclusion

Understanding the subcellular localization of lncRNA can provide great insights into their function within the cell. Computational tools have recently expanded the domain of subcellular localization by the development of faster and more accurate methods. In this study, we used a variety of machine learning as well as large language models to accurately predict lncRNA subcellular localization. The implementation of large language models to tackle biological problems is gaining momentum and our study also highlights its importance. The final model used in CytoLNCpred was designed using a traditional machine learning model trained using correlation-based features. This tool will help researchers to improve the functional annotation of lncRNA and develop RNA-based therapeutics.

## Data Availability

Publicly available datasets were analyzed in this study. This data can be found here: https://webs.iiitd.edu.in/raghava/cytolncpred/, https://github.com/raghavagps/cytolncpred.
